# Impact of predation on the bacterial community structure of Mediterranean mussels during depuration

**DOI:** 10.3389/fmicb.2025.1647926

**Published:** 2025-11-06

**Authors:** Giuseppe Blaiotta, Ivan Ciliberti, Maria Aponte, Raffaele Romano

**Affiliations:** Department of Agricultural Sciences, University of Naples Federico II, Portici, Italy

**Keywords:** *Mytilus galloprovincialis*, bacterial predation, depuration, *Vibrio mediterranei*, HTS

## Abstract

The Mediterranean mussel (*Mytilus galloprovincialis*) is the most valuable shellfish farmed and consumed in the Western Mediterranean. Like any other filter-feeding organism, mussels are exposed to a wide range of microorganisms. Before consumption, bivalves are subject to depuration to purge the gastrointestinal content, thus minimizing the risk of pathogens’ circulation. Over time, this strategy revealed several shortcomings, most notably concerning *Vibrio* spp. In this study, the potential use of autochthonous predatory bacteria as a biocontrol strategy to mitigate *Vibrio* spp. overgrowth in mussels during depuration was evaluated. Moreover, a polyphasic approach based on conventional and culture-independent strategies was used to assess the impact of predation and of depuration on the mussel microbiome during controlled depuration studies. The depuration greatly impacted the bivalve microbiota, jeopardizing its innate resilience. Moreover, the addition of a bacterial predator strain to mussels resulted in the disturbance of the microbiome. Therefore, even though the biotechnological application of bacterial predation in this context may appear promising when monitored by culture-dependent methods, the effect on the mollusks’ microbiome does not seem to be easily predictable, above all when mussels are subject to depuration after the harvest.

## 1 Introduction

The Mediterranean mussel (*Mytilus galloprovincialis*) is the most valuable shellfish produced and consumed in the Western Mediterranean. The Gulf of Naples is among the most important production sites for this type of seafood in southern Italy (Santoro et al., 2020). As filter-feeding organisms, marine mussels are constantly exposed to a wide range of microorganisms, including pathogenic bacteria that can endanger their survival. Additionally, agricultural runoff and sewage effluent contamination of coastal waterways can increase the level of shellfish microbial and viral contamination (Sharp et al., 2021). Like all macro-organisms, mussels interact not only with exogenous bacteria but also with their microbiota. This exchange between the host and its microbiota can support the preservation of their integrity (Cheikh et al., 2024).

Depuration is a controlled process that relies on the ability of bivalves to purge their gastrointestinal content by filtering clean seawater. Bivalves’ depuration is influenced by several factors, including temperature, salinity, the bivalve’s physiological state, the type of microorganism, the degree of microbial contamination, as well as the plant’s chemical or physical sterilizing system (Ottaviani et al., 2020). Depuration appeared to be a successful procedure to control fecal bacteria but proved to be less effective against naturally occurring *Vibrio* spp. (Baker, 2016). For this reason, it is necessary to develop complementary methods that, combined with conventional depuration methods, improve or extend the efficacy of depuration of live bivalves. Apart from chemical and physical methods, biological experimental applications mostly rely on the use of probiotics, bacteriocin-producing bacteria, and bacteriophages (Martinez-Albores et al., 2020), whilst the use of predatory bacteria has been rarely postulated. Predatory bacteria have been suggested as biocontrol agents only against *Vibrio* (*V*.) *parahaemolyticus* in mussels (Ottaviani et al., 2020), oysters (Li et al., 2011; Richard et al., 2012), and shrimp (Kongrueng et al., 2017; Lu et al., 2022).

Predatory bacteria have been increasingly recognized for their ubiquity in various environments and their significant functional potential in controlling unwanted microorganisms. Predatory bacteria are taxonomically and phylogenetically diverse (Zhang et al., 2024). The most studied groups of predatory bacteria include *Bdellovibrio* and *Bdellovibrio*-like organisms (BALOs) and myxobacteria. BALOs have similar functions to bacteriophages, but a broad prey spectrum. Moreover, BALOs may access EPS-containing biofilm structures and have so far been shown to be harmless to eukaryotic organisms, including plants and animals (Mookherjee and Jurkevitch, 2022). BALOs have been isolated from various habitats, including saltwater, freshwater, sewage, soils, sediments, and even animal guts and gills. BALOs are thought to play an important role in the environment as they may affect bacterial community structure and dynamics (Mookherjee and Jurkevitch, 2022). Although the potential use of predatory bacteria as living antibiotics in therapy has been the subject of numerous investigations, less is known about their ability to eradicate plant, animal, and food-borne diseases (Zhang et al., 2024). Additionally, like other biotic interactions, predation dynamics and outcomes are typically affected by abiotic and biotic factors. Nutrient availability, viscosity of the environment, surfactants, and diffusible signaling factors have all been shown to alter predation processes (Zhang et al., 2024). The informed application of predatory bacteria and the understanding of their functional roles and relevance in specific ecosystems requires the combination of classic culture-based approaches and culture-independent methodologies.

In the present study, marine predators were isolated and used as biocontrol agents against *Vibrio mediterranei* in mussels. Specifically, the effect of the predator on *V. mediterranei* was evaluated by using both depurated and non-depurated mussels. Furthermore, two distinct prey and predator inoculation levels were used. A polyphasic strategy based on both conventional and culture-independent techniques was adopted to monitor microbial dynamics.

## 2 Materials and methods

### 2.1 Sampling

Marine water samples were collected near the Naples coast during the autumn of 2022. The first sample (LN 40°49’30”–LE 14°18’48”) was used for the prey’s isolation, whilst the second one (LN 40°49’32”–LE 14°18’47”) was used for the predator isolation. Water samples were immediately transferred to the laboratory and analyzed within 1 h. pH and water conductivity were evaluated by a pH-meter (Model pH50 Lab).

### 2.2 Prey isolation and identification

Marine water was directly spread on Thiosulfate-Citrate-Bile Sucrose Agar (TCBS – Oxoid, Basingstoke, United Kingdom) plates and incubated overnight at 32 °C. At the same time, marine water was subjected to enrichment according to the protocol proposed by Huq et al. (2012). Briefly, marine water (50 mL) was added to 450 mL of Alkaline Peptone Water (APW) 10X (100 g/L peptone, 100 g/L NaCl, pH 8.6). After an overnight incubation at 32 °C, 100 μL of the top layer was spread plated onto TCBS agar plates. Colonies were randomly selected and, after repetitive streaking onto TCBS plates, cultures were used to inoculate ATCC medium n. 1,286 (Seawater complete), Tryptic Soy Broth (TSB), and Luria Bertani (LB) broths to point out the most suitable medium for cultivation. Specifically, both LB and TBS were supplemented with 20 and 30 g/L of NaCl, respectively.

A total of 10 prey isolates were identified by 16S rRNA sequencing using PCR conditions and primers - fD1 (forward, 5′-AGAGTTTGATCCTGGCTCAG-3′) and rP2 (reverse, 5′-ACGGCTACCTTGTTACGACTT-3′) - described by Weisburg et al. (1991). DNA was extracted through the protocol described by Wang et al. (1993). PCR amplicons were purified by QIAquick Gel Extraction Kit (Qiagen). The DNA sequences were determined by the dideoxy chain termination method (Sanger et al., 1977) by using the forward primers (fD1) described by Weisburg et al. (1991). Research for DNA similarity was performed with the National Centre of Biotechnology Information GenBank (Altschul et al., 1997).

### 2.3 Host preparation

Three potential prey were tested: *V. mediterranei* VM6 and *Citrobacter* (*C.) portucalensis* VM2 isolated during this study, plus one strain of *Escherichia coli* 32 isolated from meat during a previous survey. Overnight cultures of *V. mediterranei* and *C. portucalensis* in modified LB broth (20 g/L NaCl) and of *E. coli* 32 in LB (10 g/L NaCl) were centrifuged (6,500 rpm for 15 min), and the cell pellets were used for the predator isolation.

### 2.4 Predator isolation, cultivation, and identification

The enrichment protocol described by Jurkevitch (2006) was followed for the marine BALOs. Cell pellets of the prey prepared as described in paragraph 2.3 were resuspended in 100 mL of marine water to obtain a prey concentration around 10^9^/10^10^ cell/mL based on CFU counts. During incubation at 28 °C under constant stirring (Orbital shaker 300 rpm), cultures were daily monitored by spectrophotometry (600 nm) and microscope observation. Enrichments were then filtered (Minisart 0.45 μm) and, after decimal serial dilutions in sterile marine water, plated by the double-layer technique according to Jurkevitch (2006) in Pp medium (0.5 g/L tryptone, 0.5 g/L proteose-peptone, pH 7.7). Pp bottom (1.5% agar) and Pp top (1.95% agar) media were prepared in sterile marine water obtained by autoclaving water after a 0.45 μm filtration. Plates were sealed and incubated at 28 °C for 7 days. Lytic plaques were excised and resuspended in 500 μL of HM buffer [N-(2-Hydroxyethyl)-piperazine-N’-(2-ethanesulfonic acid) sodium salt] at pH 7.5. After agitation for a few minutes, the liquid containing the released BALOs was serially diluted in HM buffer up to 10^–4^.

Plaque purification was carried out according to Jurkevitch (2006) with some modifications. Cell pellets of the prey were diluted in HM buffer up to a concentration of 5 × 10^9^ cells/mL. Pp bottom (1.5% agar) and Pp top (0.7% agar) media were prepared in artificial seawater (ASW) according to Ettensohn et al. (2004). A total of 400 μL of the prey was added to 5 mL of Pp top and 100 μL of each sample’s dilution. Plates were incubated at 28 °C and monitored daily.

Predator enrichments were obtained by transferring single plaques in tubes containing the prey resuspended in 20 mL of ASW. The prey without the plaque served as a control. After 24, 48, and 72 h of incubation at 28 °C under constant shaking, tubes were monitored by spectrophotometer and microscope observation. The prey population level was assessed by the drop method (Collins et al., 1989) on modified LB agar plates. After dilution (10^1^ to 10^6^) in sterile Ringer’s solution (Oxoid), 12 μL aliquots were dropped onto agar plates using a pre-calibrated 20 μL micropipette. After incubation, individual colonies in drop areas were counted. The test allowed for obtaining indications about predatory efficiency.

For the identification of predators, enrichments were filtered (0.45 μm), and DNA was extracted by the protocol described by Wang et al. (1993). The PCR for *Bacteriovoracaceae* with primers Bac676F (forward, 5′- ATTTCGCATGTAGGGGTA-3′) and Bac1442R (reverse, 5′-GCCACGGCTTCAGGTAAG-3′) described by Davidov et al. (2006) was carried out according to the protocol detailed by the authors.

### 2.5 Strain BV5 application in mussel depuration

#### 2.5.1 Experimental plan

Mussels (*Mytilus galloprovincialis*) not depurated and still with socks, coming from the Campania Region (Italy) coasts (FAO area 37.1.3), were used for the first experiment. Mussels were sorted in the laboratory to obtain 100 individuals of the same size range (mean size = 5.83 cm ± 0.72 SD). The static method for depuration as described by Chinnadurai et al. (2023) was followed. Four batches of 20 healthy adult mussels were transferred to vessels containing one Liter of ASW for a single individual. Vessels were kept at 17 °C, and water was constantly aerated. Bivalves were not washed before immersion in ASW and were not fed during the depuration.

#### 2.5.2 Prey and predator inocula

Overnight cultures of *V. mediterranei* VM6 in Luria-Bertani NaCl-added (2%) were centrifuged, resuspended in ASW, and used as inoculum up to a final population level of about 10^2^ CFU (Colony-Forming Units)/mL. Before the predator was introduced, mussels were allowed to gather the prey for 5 h (Ottaviani et al., 2020).

The enrichment to be used as inoculum for the predator was prepared by transferring a plaque in 80 mL of a cell suspension of *V. mediterranei* in ASW. The suspension was obtained by centrifuging an overnight culture (20 mL) of the prey in Luria-Bertani with NaCl (2%). After 48 h of incubation at 28 °C under stirring, the enrichment was filtered (Minisart 0.45 μm) to remove the prey and used for inoculation at a level of about 10^6^ PFU (Plaque-Forming Unit)/mL.

#### 2.5.3 Experimental plan

**FIGURE 1:**
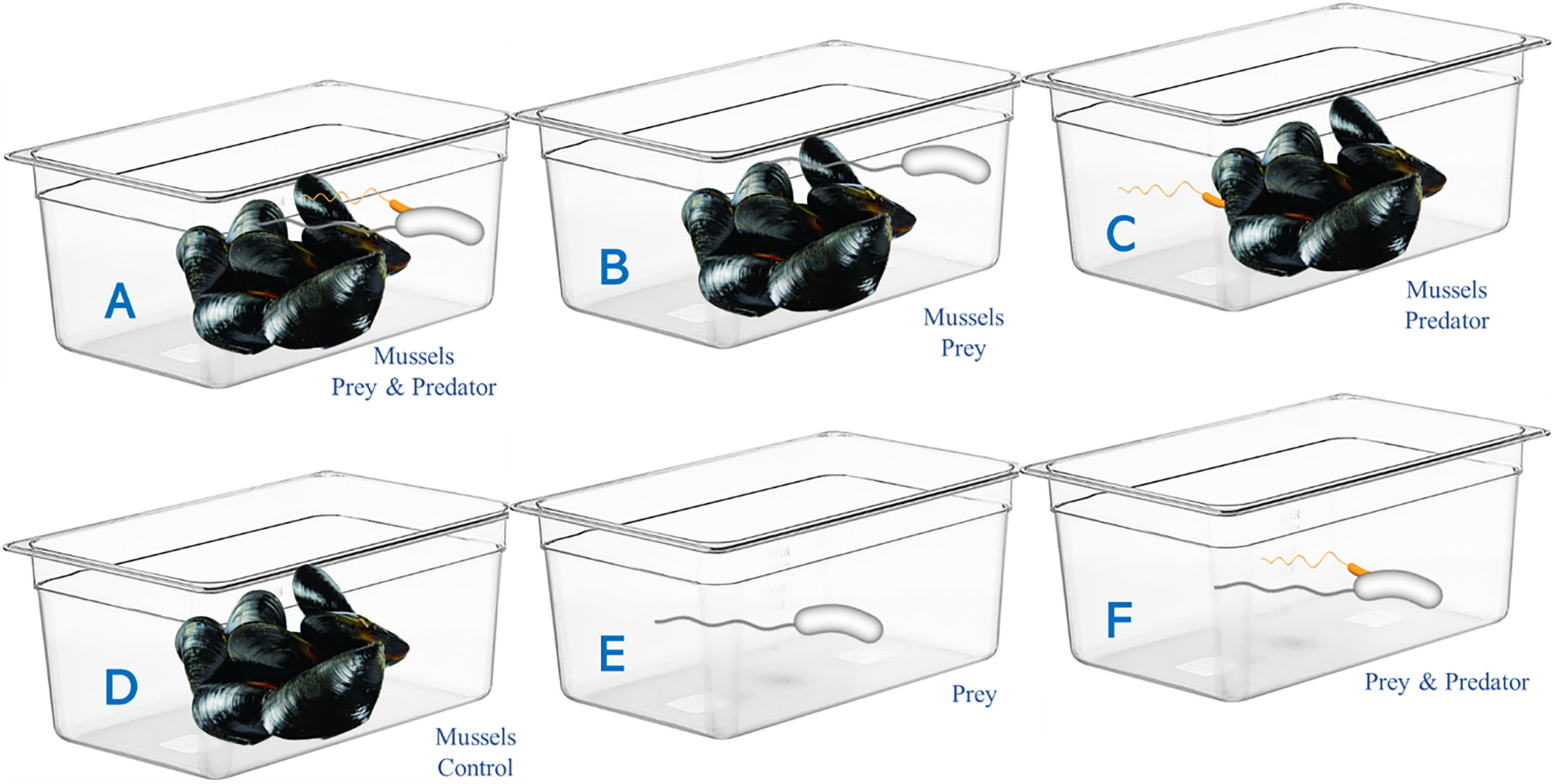
Experimental design used for the depuration. **(A)** Mussels inoculated with prey (*V. mediterranei* VM6) plus predator (BV5); **(B)** Mussels plus *V. mediterranei* VM6; **(C)** Mussels plus BV5; **(D)** Mussels control; **(E)** (Only prey) and **(F)** (Prey and predator) in artificial seawater (ASW) without mussels.

The experimental plan may be schematized as follows: Trial (A) Not depurated mussels plus prey (strain VM6) plus predator (strain BV5); Trial (B) Not depurated mussels plus prey (VM6); Trial (C) Not depurated mussels plus predator (BV5); Trial (D) Not depurated mussels (control). Moreover, two further trials were carried out in ASW without mussels, and specifically, Trial (E) hosted the sole prey (VM6), while Trial (F) included both prey (VM6) and predator (BV5). The experimental design is detailed in Figure 1.

The same experiment, organized in six distinct trials, was repeated by using depurated mussels coming from the same farm and the prey at a higher level of inoculum (10^4^CFU/mL).

#### 2.5.4 Microbial populations monitoring by culture-dependent methods

Mussels, in the adult stage and of similar size, were used for microbial counts and DNA extraction. Bivalves were scrubbed to remove epibionts, opened with a sterile knife, and the whole content (digestive gland, gills, foot, mantle, and liquid) was placed in a stomacher bag. At time 0, and after 5, 24, and 48 h, at least three mussels from each trial were collected for microbial counts. ASW was monitored as well. At each sampling point, heterotrophic bacteria were counted on Water Plate Count agar (WPCA) after incubation at 22 °C for 3 or 7 days. *Enterobacteriaceae* and coliforms were monitored on Violet Red Bile Glucose agar (VRBGA) and Violet Red Bile Lactose agar (VRBLA), respectively, in both cases after incubation at 37 °C for 24–48 h in microaerophilic conditions generated by the double layer technique. *Vibrionaceae* were enumerated on TCBS after incubation at 37 °C for 24 h, Enterococci and *E. coli* on Slanetz & Bartley (SB) and Tryptone Bile X-Gluc Medium (TBX), respectively, in both cases after incubation at 37 °C for 24–48 h. Analyses were performed in duplicate. All media and supplements were provided by Oxoid.

During the first experiment, predators were monitored at each sampling point uniquely in ASW by the plaques forming method described in “see section 2.3 Host preparation.” Conversely, in the second experiment, predators were searched in mussels from trial A, too.

#### 2.5.5. High-throughput sequencing (HTS) analysis of bacterial communities

The microbiome of mussels and waters after 24 and 48 h of depuration by trials A, B, C, and D of the first and the second set of experiments (Depurated and Not-depurated mussels) was monitored by HTS. Mussel samples for total DNA extraction (about 200 mg) were collected as described in “see section 2.5.4 Microbial populations monitoring by culture-dependent methods.” For water samples, an amount of 200 mg of wet biomass was gathered by sequential centrifugations at 14,000 × *g* for 10 min at 4 °C. In both cases, DNA extraction was carried out by using the NucleoSpin^®^ Food Kit (Macherey-Nagel, Düren, Germany). Bacterial communities were assessed by HTS of the amplified V3–V4 regions within the 16S rRNA gene (∼460 bp). PCR was carried out with primers (S-D-Bact-0341-b-S-17/S-D-Bact0785-a-A-21) connecting with barcodes (Aponte et al., 2022). PCR products with the proper size were selected by 2% agarose gel electrophoresis. The same amount of PCR products from each sample was pooled, end-repaired, A-tailed, and further ligated with Illumina adapters. The library was checked with Qubit and real-time PCR for quantification and bioanalyzer for size distribution detection. Quantified libraries were pooled and sequenced on a paired-end Illumina platform Novaseq PE250, to generate 250 bp paired-end raw reads. Paired-end reads were joined by using FLASH (Magoč and Salzberg, 2011). The DADA2 method (Callahan et al., 2016) was used for noise reduction. ASVs (Amplicon Sequence Variants) were further filtered by using QIIME2 software (Version QIIME2-202202) and identified by using the Silva Database 138.1. Unassigned sequences and those assigned to eukaryotes (i.e., chloroplasts and mitochondrial ones) were discarded. Statistical analyses and plotting were carried out in R environment.^1^ Shannon and Simpson alpha-diversity indices were calculated through the function “diversity.”

Furthermore, upon arrival, 10–12 mussels in the adult stage, either depurated or not, were pooled and subject to DNA extraction by using the NucleoSpin^®^ Food Kit and analyzed by HTS.

### 2.6. Statistical analysis

Results of CFU, PFU, and OD600 values were expressed as mean ± standard deviation. Significant differences among data were computed by using ANOVA and Tukey *t*-test (*p* < 0.05) (XLStat 2012.6.02 statistical pocket, Addinsoft Corp., Paris, France).

## 3 Results

### 3.1 Prey isolation and identification

Based on the colonies’ appearance on TCBS agar plates seeded with marine water (pH 7.91 ± 0.01; conductivity −52 ± 0.01 mV) and from plates seeded with the enrichments, ten colonies in total were chosen. According to the results obtained by the 16S rRNA partial sequencing, one strain could be reported to *V. mediterranei* (99% similarity), two to *Photobacterium* spp. (98% similarity with *Photobacterium* sp. strain 7–11 KX806606.1 in both cases), two to *C. portucalensis* (99 and 100% similarity with *C. portucalensis* strain 68soilLBA LC717361.1), three to *V. harveyi* (98 or 99% with *V. harveyi* strain B8-1MK102617.1), and two to *V. chagasii* (99% with *V. chagasii* strain GCZ10 MH613265.1).

### 3.2 Predator isolation and identification

Upon enrichment with the three prey (*V. mediterranei* VM6, *C. portucalensis* VM2, and *E. coli* 32), the faster clarification was noticed only against the unique strain of *V. mediterranei* (Data not shown). At the microscope, small, speedy motile cells could be observed. Five lysis plaques were purified. None of the five strains - BV1, BV2, BV3, BV4, and BV5 – generated an amplicon by the family-specific PCR, so despite the morphological and physiological similarities with BALOs, strains isolated during this study cannot be considered members of this group.

### 3.3 Evaluation of predatory efficiency

Cultures BV1, BV2, BV3, BV4, and BV5 exhibited a rather variable predatory efficiency. Strain BV4 proved to induce a significant decrease in OD values at 24 (1.841 ± 0.002) and 48 h (1.149 ± 0.001); strain BV5 induced the highest clarification at 48 h (0.997 ± 0.001). Moreover, this strain produced a significant decrease in *V. mediterranei* CFU/mL at 48 h (from 9.00 ± 0.23 to 7.73 ± 0.09 CFU/mL) (Supplementary Figure 1). Based on results, strain BV5 was selected for further experiments.

### 3.4 BV5 application in mussel depuration

First, the growth of *V. mediterranei* strain VM6 on TCBS was compared with that on WPCA and TSA media. The counts levels on TCBS and TSA were equal, whereas it was ascertained that the prey was unable to grow on WPCA, thus proving that counts on this medium could not be affected by the prey inoculum.

**FIGURE 2:**
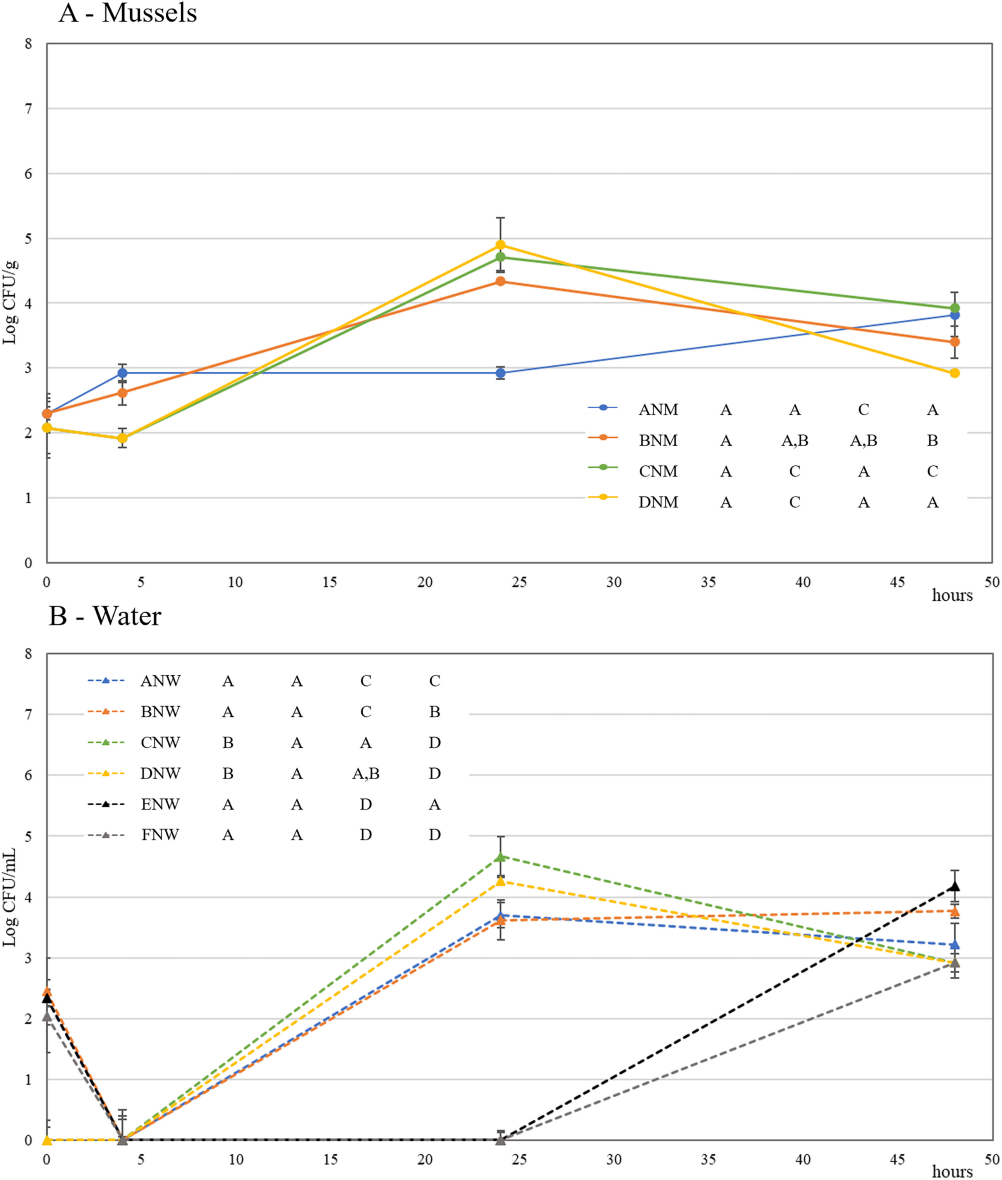
Mussel depuration, experiment 1. Dynamics of *Vibrio* spp. counts (Log CFU/mL or gr ± sd) on TCBS in non-depurated mussels (NM – Panel **A**) and waters (NW – Panel **B**) at 0, 5, 24, and 48 h. ANM and ANW: *V. mediterranei* VM6 plus BV5; BNM and BNW: only VM6; CNM and CNW: only BV5; DNM and DNW: mussel control. ENW and FNW: prey and prey plus predator in ASW without mussels. For data with the same letter, differences between trials are not statistically significant (*p* < 0.05).

In the first experiment, mussels not previously subject to depuration were used. The prey inoculum was fixed at about 10^2^ CFU/mL in trials A, B, E, and F. *Vibrionaceae* were monitored on TCBS. Still, specifically, only yellow colonies similar to those produced by *V. mediterranei* VM6 on this medium were selectively counted. *Vibrio* populations in mussels were in all trials (A–D) in the range of 10^2^–10^3^ CFU/mL, namely, the same adopted for the prey inoculation in this experiment (Figure 2A). However, by comparing TCBS counts in mussels collected by trials A and B, namely those inoculated with BV5 plus VM6 and strain VM6 alone, respectively, an interesting outcome may be pointed out: after 24 h, the decline in *Vibrio* populations in trial A, which included both prey and predator, was greater than one Log. Such difference disappeared after 48 h, likely as a result of the prey growth due to nutrients released by mussels (Figure 2A).

In trial C, the predator addition did not induce any changes, and this might be linked to the high specificity of the strain BV5 regarding the prey. As a general consideration, *Vibrionaceae* increased by more than one Log in the control (Trial D).

The monitoring of *Vibrionaceae* in the depuration water showed a rather different trend, and the difference between trials A and B became statistically significant only after 48 h of purification (Figure 2A). In the control (Trial D), vibrios grew exponentially, demonstrating that a transfer from mussels into water occurs during depuration. The monitoring of the prey (Trial E) and of prey plus predator (Trial F) in ASW without mussels confirmed the predatory efficiency of the strain BV5: *Vibrio* reduction after 48 h was higher than one Log (Figure 2B).

Predators were monitored by PFU counts in water collected from trials A, C, and F (Supplementary Figure 2). Populations increased by more than one Log after 48 h in trial F, namely when prey and predator were alone in sterile ASW, stayed constant in trial C (predator inoculated into mussel depuration water), and slightly decreased in trial A despite the prey presence.

Heterotrophic microflora was monitored in both water and mussels (Supplementary Table 1). Counts were almost stable in mussels, whilst a slight decrease characterized counts in water. The high contamination level in water, around 3 Log CFU/mL, could be due to the high bacterial release from mussels that were not washed and still with the socket. Despite not being depurated, *Enterobacteriaceae* on VRBGA, *Coli-aerogenes* group on VRBLA, *E. coli* on TBX, and enterococci on SB were undetectable throughout the monitoring (Supplementary Table 1).

**FIGURE 3:**
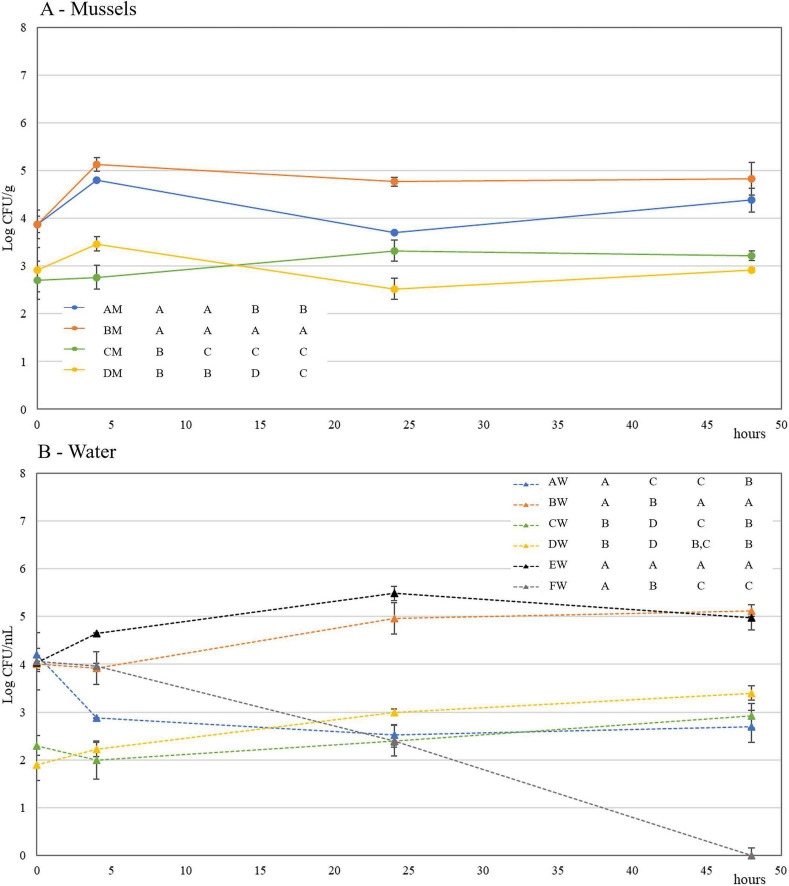
Mussel depuration experiment 2. Dynamics of *Vibrio* spp. counts on Thiosulfate-Citrate-Bile Sucrose Agar (TCBS) in depurated mussels [M - **(A)**] and waters [W – **(B)**] at 0, 5, 24, and 48 h Trials are described in Figure 1. For data with the same letter, differences between trials are not statistically significant (*p* < 0.05).

The same set of experiments was repeated by using mussels already depurated and a higher level of inoculum for the prey (about 4 Logs CFU/mL of water). Despite depuration, the level of countable yellow colonies on TCBS was around 2 Log CFU/mL in trials C and D carried out without the prey addition (Figure 3A). However, in this case, the adoption of a higher level of inoculum for *V. mediterranei* allowed the discrimination of the prey from the naturally occurring vibrios in mussels. In fact, in trials A and B, after 5 h, the level of vibrios in mussels was almost one Log higher than trial C (only the predator added), and control trial D (Figure 3A).

Also in this depuration experiment, the maximum predation occurred after 24 h, and, exactly as for the first set of experiments, the BV5 inoculum in trial C did not affect the autochthonous *Vibrio* population (Figure 3A).

In the water, a significant drop in *Vibrio* population can be linked to predation: the difference in *Vibrio* loads between trials A and B at 24 h was higher than two Logs (Figure 3B). In trials E and F, carried out without mussels, the outcomes were even more remarkable: after 48 h, *V. mediterranei* counts dropped below the method’s detection limit. PFU monitoring confirmed the evidence collected during the first experiment: predators in water from trials in which the strain BV5 was added did not change in number (Supplementary Figure 3A). As expected, plaques were not retrieved in trial E, containing only the prey in ASW, whereas in trial B (prey and mussels), as well as in trial D – the control – autochthonous predators were found despite being undetectable at time 0 (Supplementary Figure 3A). The level reached was around 3 Log PFU/mL. Furthermore, in the unique case of trial A, predators were monitored in mussels as well: after 24 h, a noticeable predator concentration inside mussels was evidenced (Supplementary Figure 3B).

Water Plate Count agar counts were by two Logs higher in depurated mussels and stayed almost stable in both mussels and ASW in all trials, thus proving that the presence of strain BV5 does not affect the naturally occurring microflora in both environments. Despite being depurated, mussels hosted *Enterobacteriaceae* and coliforms, whereas enterococci were only seldom detected (Supplementary Table 2). In mussels from trial B and control sample D, blue colonies on TBX revealed a low occurrence of *E. coli* (Supplementary Table 2).

### 3.5 Microbial dynamics in mussels and water by HTS

16S rRNA gene amplification and amplicon sequencing were successful for all samples. A total of 1,381,261 classified reads were obtained. The mean number of reads per sample was 38,368 ± 15,897. The phylum *Campylobacterota* was the most abundant in mussels from the experiment carried out with non-depurated mussels; conversely, in bivalves already subject to depuration, a higher biodiversity could be noticed (Supplementary Figure 4). In water samples, *Proteobacteria* (now *Pseudomonadota*) were prevalent, especially in the second set of the experiment. Subdominant phyla were *Firmicutes* (now *Bacillota*), *Bacteroidota*, *Fusobacteriota*, and *Patescibacteria*. The abundance of *Bdellovibrionota* was significant only in water samples from non-depurated mussels and not exclusively in predator-added trials (Supplementary Figure 4).

**FIGURE 4:**
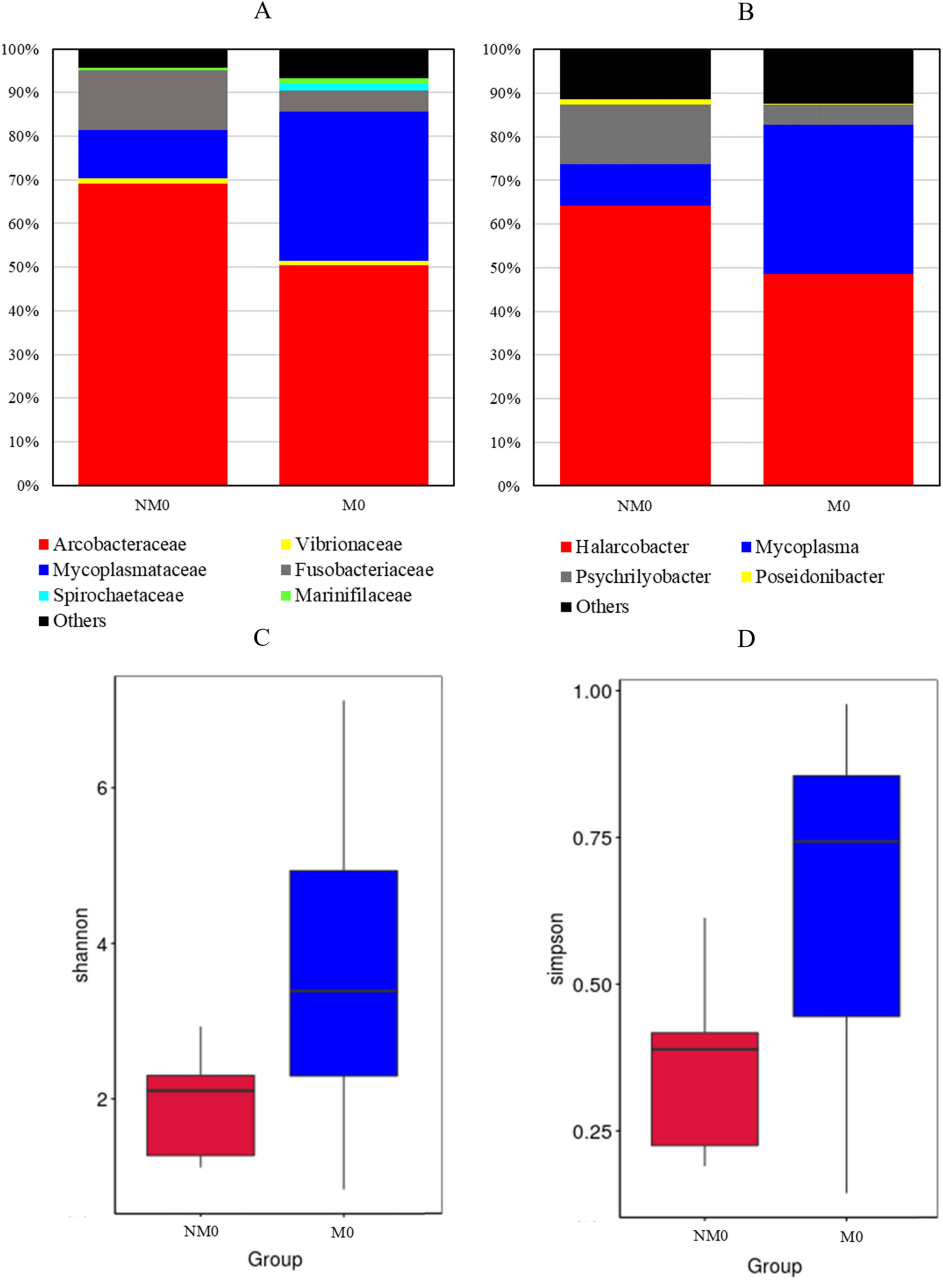
Barplots showing the mean relative abundance of bacterial families in non-depurated (NM) and depurated (M) mussels and relative waters (NW and W). The first letter of the code refers to the trial **(A–D)**, and the final number indicates the sampling time (24 and 48 h). Only taxa with a mean relative abundance > 1% are plotted.

The phylum *Campylobacterota* was essentially represented by species within the family *Arcobacteraceae* in trials carried out with non-depurated mussels (Figure 4A); conversely, in depurated mussels, a higher variability could be recorded, above all in samples where strain BV5 was added (Trials A and C): after 48 h, a noticeable increase in the *Bacteroidaceae*, *Lachnospiraceae*, and *Ruminococcaceae* occurrence could be observed (Figure 4B).

Moreover, in the first set of trials, the presence of *Vibrionaceae* was almost identical in all trials (Figure 4A). As already evidenced by microbial count, the adjunct of 2 Log/mL of *V. mediterranei* VM6 was hidden by the occurrence of autochthonous vibrios at about the same population level. In mussels from trials carried out by using a higher inoculum level for the prey, the relative abundance of *Vibrionaceae* in mussels increased with time in both trials A and B (Figure 4B): in other terms, the predator’s adjunct does not seem to control the *Vibrio* accumulation due to mussels’ filtration (Supplementary Figure 3). Indeed, the abundance of *Vibrionaceae* in mussels increased over time in the control (Trial D) as well (Figure 4B).

Concerning water, the relative abundance of *Vibrionaceae* markedly decreased in trials A of both depuration experiments (Figures 4C, D). The family *Bacteriovoracaceae* was detected in all samples. The relative abundance increased by passing from 24 to 48 h in almost all trials, independently of the type of mussels used for the experiments.

*Enterobacteriaceae* appeared to be more represented in depurated mussels and, in trial A, after 24 h, this family dominated the microbiota (Figure 4B). Such an outcome corroborated results obtained by counting on selective media.

*Arcobacteraceae* were, as expected, dominant in water coming from trials with non-depurated mussels (Figure 4C), whereas, in the water of experiments with depurated mussels, *Pseudoalteromonadaceae* exhibited the highest relative abundance (Figure 4D).

**FIGURE 5:**
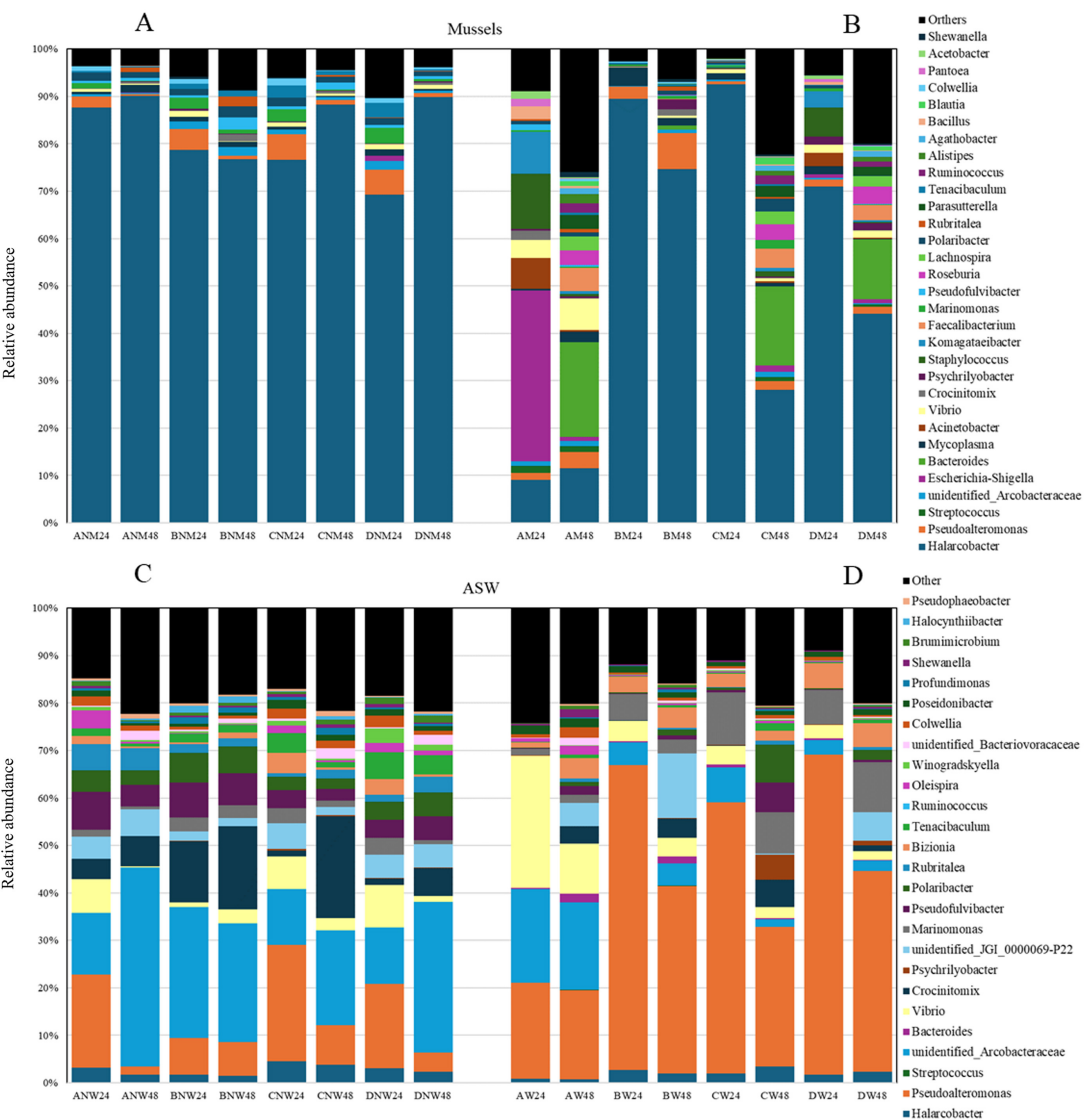
Barplots showing the mean relative abundance of bacterial genera in non-depurated (NM) and depurated (M) mussels **(A,B)** and depuration waters (NW and W in **C, D**) at 24 and 48 h of depuration. Sample codes are detailed in the caption of Figure 3. Only taxa with a mean relative abundance >1% are plotted.

By passing to genera, the percentage of reads that could not be reported to any taxa ranged from 2.04 to 25.93% and from 8.92% to 24.19% for mussels and waters, respectively (Figure 5). In non-depurated mussels, the genus *Halarcobacter* spp. dominated (Figure 5A). The genus *Pseudoalteromonas* spp. – was prevalent in depurated mussels (Figure 5B).

The genera *Mycoplasma*, *Polaribacter*, and *Rubritalea* spp. were detected in all mussel samples with relative abundances in the range 0.26–3.84, 0.15–2.63, and 0.02-2.17, respectively. Concerning the genus *Vibrio* spp., in the first set of experiments, the low level of the inoculum did not allow for highlighting any change. The relative abundance in trial A after 24 h is comparable with that of the control (Figure 5A). The second set of experiments, with a higher inoculum level, allows some considerations to be inferred. The relative abundance of vibrios increased by passing from 24 to 48 h in mussels from trial A with non-depurated mussels, allowing to confirm that a vibrios migration in bivalves takes place during depuration (Figure 5B). In waters, the relative abundance of the *Vibrio* genus was noticeably high only after 24 h in trial A, and, in all cases, the abundance of this genus decreased after 48 h (Figure 5C).

In waters, only one BALOs was detected and was reported as unidentified *Bacteriovoracaceae* by HTS. The relative abundance was quite low in water from both sets of experiments, regardless of the trial. Nevertheless, the relative abundance significantly increased by passing from 24 to 48 h in ASW from trial A, where the strain BV5 was inoculated together with the prey (Figure 5C).

### 3.6 Effect of commercial depuration on mussel microbiome

**FIGURE 6:**
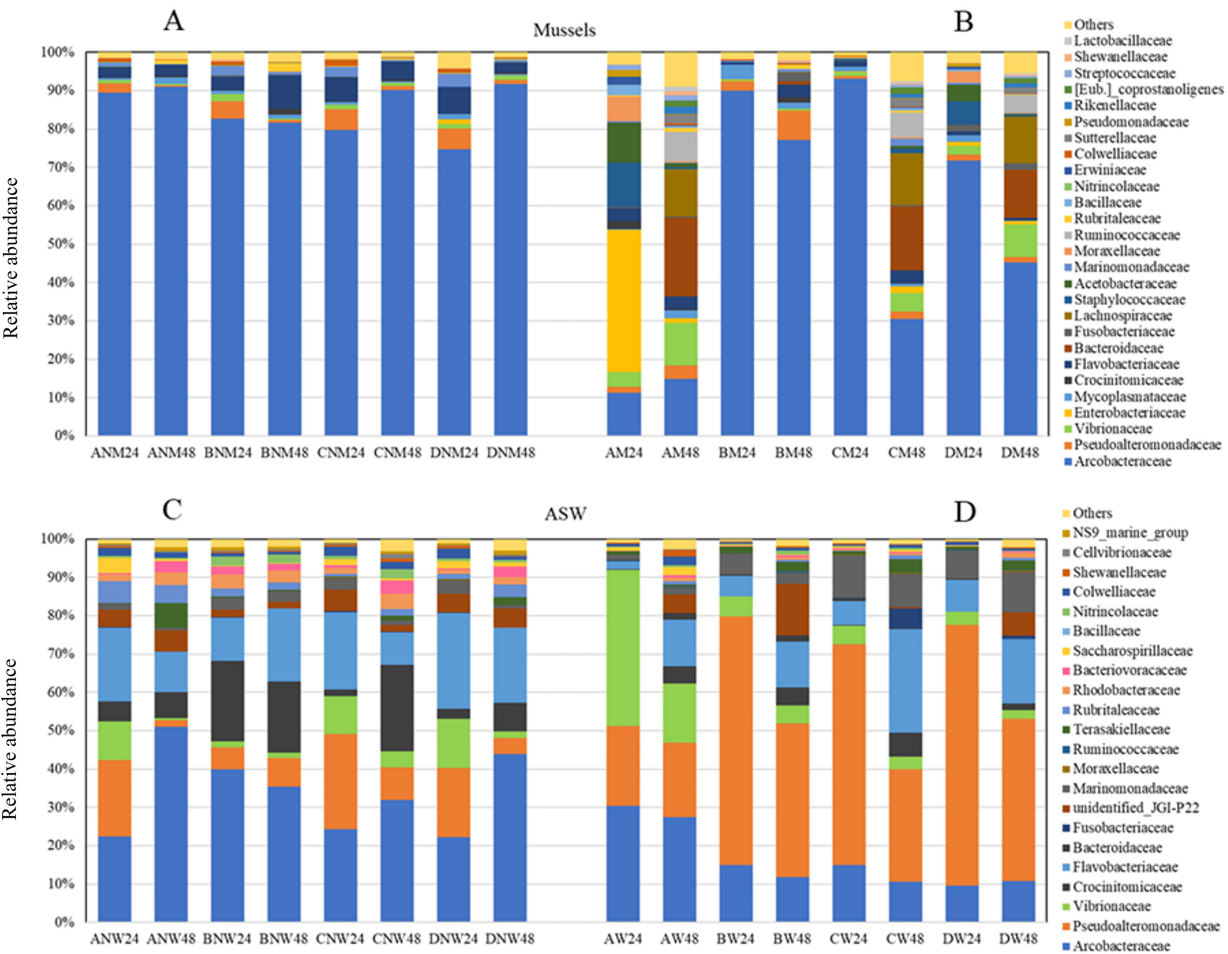
Barplots showing the mean relative abundance of bacterial families **(A)** and genera **(B)** in non-depurated (NM0) and depurated (M0) mussels upon arrival. Alpha diversity boxplot of NM0 and M0 bacterial communities based on the Shannon **(C)** and Simpson **(D**) indices. Only taxa with a mean relative abundance > 1% are plotted.

The microbiome of depurated and non-depurated mussels was analyzed by HTS (number of reads 50,957 and 42,458, respectively). Distribution appeared to be rather different at both family and genus levels (Figures 6A, B). In mussels that were not previously depurated, *Arcobacteraceae* were dominant, followed by *Fusobacteriaceae* and *Mycoplasmataceae*. In depurated mussels, *Mycoplasmataceae* were almost equally represented as *Arcobacteraceae*.

At the genus level, apart from *Halarcobacter* and *Mycoplasma* - which were retrieved in mussels subject to depuration in the lab as well, it is remarkable the relative abundance of the *Psychrilyobacter* spp. This genus was instead poorly represented in all mussel samples coming from trials A, B, C, and D.

The alpha diversity analysis, especially the Simpson’s diversity index, revealed a higher biodiversity in depurated mussels (Figures 6C, D).

## 4 Discussion

### 4.1 Selection of prey and predator

*V. mediterranei* VM6 was selected as prey since it is more susceptible to lysis than the *C. portucalensis* VM2 and *E. coli* 32, regardless of the predator. The lytic capabilities of bacterial predators against hosts may greatly vary, resulting in wildly disparate prey spectrum ranges. While some predators exhibit large ranges that cover numerous Gram-negative and even some Gram-positive bacteria, others have very narrow ranges, covering only a few species or strains (Najnine et al., 2020). The species *V. mediterranei* has been recognized as a pathogen of the razor clam - *Sinonovacula constricta* - (Fan et al., 2023), and of the noble pen shell - *Pinna nobilis* - a large bivalve on the brink of extinction in the Mediterranean (Andree et al., 2021). Recently, this species has been demonstrated to provoke significant acute immune responses and tissue-level reactions in *M. galloprovincialis* (Ter et al., 2024). Furthermore, the presence of a strain-specific pathogenicity island was established by the comparative investigation of 21 strains of *V. mediterranei*, underscoring the species’ pathogenicity toward bivalves (Zhang et al., 2025). From this angle, in addition to the hypothesized control of *Vibrio* populations during depuration, the one-log reduction of *V. mediterranei* VM6 ensured by the strain BV5 after 48 h could prevent larval vibriosis in the shellfish farming.

### 4.2 Impact of the predator and laboratory-scale depuration on the mussel microbiome

In all depuration experiments, *Vibrionaceae* naturally occurring in mussels were around 10^2^–10^3^ CFU/mL. Several authors have already reported a prevalence of *Vibrio* spp. in seafood samples collected in Italy (Parisi et al., 2004; Serracca et al., 2007; Sferlazzo et al., 2018). Moreover, during depuration, *Vibrionaceae* increased by more than one Log in the control (trial D), thus confirming that, as previously reported (Serratore et al., 2014; Sferlazzo et al., 2018), the purification treatment, worldwide utilized to purge bivalve mollusk from fecal contaminants, is unsatisfactory for seawater autochthonous *Vibrio* spp. These results might be related to the vibrios’ release dynamics by bivalves in depuration (Suffredini et al., 2014). At any rate, during the second set of trials carried out with a higher level of prey inoculum, the predator addition provided satisfactory outcomes. Indeed, prey concentration is well-known to influence the efficacy of BALOs’ predation. Specifically, predatory activity seems to be inhibited at prey levels below 10^4^ CFU/mL (Williams et al., 2016). Results were in agreement with those reported by Ottaviani et al. (2020) for the *Halobacteriovorax* sp. strain HBXCO1: the predator at 10^3^ PFU/mL was able to keep 10^5^ CFU/mL of *V. parahaemolyticus* to about 2 Logs lower than that of the control during mussels’ depuration.

During the experiment conducted with non-depurated mussels, the predators monitoring in the water revealed that populations increased only in trial F, namely when prey and predator were alone in sterile ASWF (Supplementary Figure 2). This discrepancy might be explained by the predator migration inside mussels. In fact, PFU monitoring validated the results when the experiment was conducted again using depurated mussels. After a day, the number of predators in trial A mussels increased, supporting the theory that predators migrate into mussels (Supplementary Figure 3B).

By HTS, the phylum *Campylobacterota* was the most abundant in non-depurated mussels (Supplementary Figure 4). Results did not match those reported for Australian *M. galloprovincialis* mussels. According to Odeyemi et al. (2019), the three major phyla in the mussel meat and pouch water of non-depurated and depurated mussels were *Proteobacteria*, *Cyanobacteria*, and *Firmicutes*. *Proteobacteria*, *Tenericutes*, and *Bacteroidetes* are indicated as dominant taxa at the phylum level in Greek mussels in both winter and summer months by Schoinas et al. (2023).

More info can be obtained by analyzing the families’ dynamics in the four trials and during the time (Figure 4). *Arcobacteraceae* dominated the microbiome of non-depurated mussels, whilst depurated mussels were characterized by a higher variability, above all in trials with the predator: *Bacteroidaceae*, *Lachnospiraceae*, and *Ruminococcaceae* increased along time (Figure 4B). The mussel microbiota appeared to include well-known animal microbial commensals involved in carbohydrate oxidation or fermentation and are likely able to influence the gastrointestinal metabolism of the host, as demonstrated in terrestrial animals. Indeed, the description of mussel microbiome on the tissue scale has revealed that the microbiota of each tissue is characterized by a specific pattern, with the digestive gland microbiota being dominated by *Ruminococcaceae* and *Lachnospiraceae*: bacteria able to ferment complex polysaccharides into short-chain fatty acids, and thus well matching the general assets of the animal gut microbiota (Musella et al., 2020).

Regardless of the trial, the abundance of *Vibrionaceae* in mussels increased over time. An expanding number of environmental studies have contributed to improving knowledge about the family *Vibrionaceae*, and some new species, such as *V. crassostreae*, *V. breoganii*, and *V. celticus*, are described as forming part of the molluscan microbiota (Romalde et al., 2014).

In non-depurated mussels, the genus *Halarcobacter* spp. dominated all samples (Figure 5). This genus is one of the six obtained by the split of *Arcobacter* spp. (Pérez-Cataluña et al., 2018). Natural inhabitants of environmental waters, including surface water, groundwater, rivers, lakes, and seawater, members of this genus have also been found in sewage and plankton (Zhang et al., 2019). The overall prevalence of *Arcobacter* spp. in bivalves has been previously reported (Laishram et al., 2016; Morejón et al., 2017; Rathlavath et al., 2017; Salas-Massó et al., 2016; Zhang et al., 2019). High percentages have been reported in Italy as well. Mottola et al. (2016) isolated *Arcobacter* spp. from shellfish samples in the Apulian region in Italy, while Fera et al. (2004) detected this genus in seawater and plankton samples collected from the Strait of Messina. The genus *Arcobacter* spp., previously known as the aero-tolerant *Campylobacter*, has gained clinical significance as an emerging diarrheagenic pathogen associated with water reservoirs in recent years. The complete clinical significance of *Arcobacter* remains rather speculative due to the virulence and antibiotic susceptibility of individual strains (Barel and Yildirim, 2023). On the other hand, the microbiome of depurated mussels appeared to be dominated by *Pseudoalteromonas* spp. This genus is widely distributed in various marine environments. Many *Pseudoalteromonas* species may induce the settlement of larvae of several invertebrates, including *Mytilus coruscus* (Wang et al., 2019).

The genera *Mycoplasma*, *Polaribacter*, and *Rubritalea* spp. detected in all mussel samples have been reported as dominant in the mussel microbiome by Schoinas et al. (2023). On the other hand, other genera reported as dominant by the authors, such as *Anaplasma*, *Ruegeria*, and *Mariniblastus* spp., were not detected in the present study.

The only BALOs family detected in waters by HTS was an unidentified *Bacteriovoracaceae*. The relative abundance in trials with both prey and strain BV5 increased over time (Figure 5C). Since genus-specific PCR did not provide amplification when tested on strain BV5, it is not reasonable to guess that the added predator was the unidentified *Bacteriovoraceae* detected by HTS. In general, the addition of the strain BV5 to depurated mussels, even in the absence of prey (Trial C), resulted in a disturbance of the microbiome at both the family and genus levels. The circumstance that mussels had previously undergone a depuration treatment may have caused a loss of the microbiome’s innate resilience, raising the risk that the bacterial communities would be altered.

### 4.3 Impact of commercial depuration on the microbiota of mussels

The mussels used for the two decontamination experiments were provided by the same supplier, and they were non-depurated for the first set of trials and already depurated for the second. Since both types of mussels were analyzed immediately upon arrival, HTS might offer insights into how commercial depuration affects the mussel microbiome. A considerable decrease in the relative abundance of *Psychrilyobacter* spp. suggests that depuration may have a major effect on the mussel microbiome (Figure 6). *Psychrilyobacter* spp. is a globally distributed bacterial genus with an inhabiting preference for the gut of marine invertebrates such as the European abalone (*Haliotis tuberculata*), regardless of the season and feeding diet, but also of oysters, sea vases, Atlantic salmon, deep-sea snails, green-lipped mussels, Chilean mussels, and even deep-sea hydrothermal vent crabs (Liu et al., 2023). In the present survey, the relative abundance of this genus decreases along with the mussels’ permanence in water, and this evidence does not seem to prove its role as a mussel holobiont. As a matter of fact, in *Mytilus chilensis*, the genus *Psychrilyobacter* spp. appeared to be dominant in mussels living in natural conditions (Santibáñez et al., 2022), and its presence could be linked uniquely to the marine sediments: an ecosystem where the genus *Psychrilyobacter* is associated as an important protein and/or amino acid degrader (Pelikan et al., 2021). Additionally, in oysters, this genus increases in flesh during the moribund peak of the Pacific Oyster Mortality Syndrome caused by Ostreid Herpesvirus 1 infection (Richard et al., 2021).

The decrease in the relative abundance of both *Psychrilyobacter* and *Halarcobacter* genera goes along with the increase of *Mycoplasma* spp. (Figure 6). Indeed, the proliferation of subdominant phyla after a depuration of 15 h has already been reported for the mussel hepatopancreas bacteriome (Rubiolo et al., 2018) and the haemolymph (Vezzulli et al., 2018).

## 5 Conclusion

The initial goal of the present study was to evaluate the potential of predators for controlling vibrios populations during mussel depuration. In fact, traditional depuration methods can significantly reduce coliforms and other transient bacteria in farmed bivalve tissues, but are only somewhat unsuccessful in eliminating other microorganisms, such as naturally occurring marine vibrios.

Based on results, the biotechnological application of predators in this context might appear promising when monitored by culture-dependent methods. Conversely, the effect on the mollusk microbiome does not seem to be easily predictable, especially when mussels have been subjected to transfer in water after the harvest. Furthermore, according to the gathered outcomes, depuration significantly affects the bivalve microbiota and may favor opportunistic members of the bacterial community. The loss of resilience of the mussel microbiome upon depuration is also revealed by the deep impact that the predator addition proved to exert on the microbial ecosystem. Such an outcome poses several criticisms of the opportunity to adopt this approach. Nevertheless, the role of natural predation during depuration has not been investigated yet, but its contribution to decontamination by Gram-negative bacteria certainly needs more attention.

## Data Availability

The 16S rRNA gene sequences are available at the Sequence Read Archive (SRA) of the National Center for Biotechnology Information (NCBI), under accession number PRJNA1298280.

## Footnotes

1
http://www.r-project.org

## References

[B1] Altschul S. F. Madden T. L. Schäffer A. A. Zhang J. Zhang Z. Miller W. (1997). Gapped BLAST and PSI-BLAST: A new generation of protein database search programs. *Nucl Acids Res.* 25 3389–3402. 10.1093/nar/25.17.3389 9254694 PMC146917

[B2] Andree K. B. Carrasco N. Carella F. Furones D. Prado P. (2021). *Vibrio mediterranei*, a potential emerging pathogen of marine fauna: Investigation of pathogenicity using a bacterial challenge in *Pinna nobilis* and development of a species-specific PCR. *J. Appl. Microbiol.* 130 617–631. 10.1111/jam.14756 32592599

[B3] Aponte M. Esposito F. Sequino G. Blaiotta G. De Filippis F. (2022). Stuck or sluggish fermentations in home-made beers: Beyond the surface. *Int. J. Food Microbiol.* 383:109956. 10.1016/j.ijfoodmicro.2022.109956 36209540

[B4] Baker G. L. (2016). Food safety impacts from post-harvest processing procedures of molluscan shellfish. *Foods* 5:29. 10.3390/foods5020029 28231124 PMC5302340

[B5] Barel M. Yildirim Y. (2023). *Arcobacter* species isolated from various seafood and water sources; virulence genes, antibiotic resistance genes and molecular characterization. *World J. Microbiol. Biotechnol.* 39:183. 10.1007/s11274-023-03547-x 37147408

[B6] Callahan B. J. McMurdie P. J. Rosen M. J. Han A. W. Johnson A. J. A. Dada S. H. (2016). High-resolution sample inference from Illumina amplicon data. *Nat. Meth.* 13 581–583. 10.1038/nmeth.3869 27214047 PMC4927377

[B7] Cheikh Y. B. Massol F. Giusti-Petrucciani N. Travers M. A. (2024). Impact of epizootics on mussel farms: Insights into microbiota composition of *Mytilus* species. *Microbiol. Res.* 280:127593. 10.1016/j.micres.2023.127593 38184970

[B8] Chinnadurai S. Elavarasan K. Geethalakshmi V. Kripa V. Mohamed K. S. (2023). Development of a depuration protocol for commercially important edible bivalve molluscs of India: Ensuring microbiological safety. *Food Microbiol.* 110:104172. 10.1016/j.fm.2022.104172 36462828

[B9] Collins C. H. Lyne P. M. Grange J. (1989). “Counting microorganism,” in *Microbiological Meth*, eds Collins C. H. Lyne P. M. Grange J. M. (Waltham, MA: Butter-worth-Heinemann), 127–140.

[B10] Davidov Y. Friedjung A. Jurkevitch E. (2006). Structure analysis of a soil community of predatory bacteria using culture-dependent and culture-independent methods reveals a hitherto undetected diversity of *Bdellovibrio*-and-like organisms. *Environ. Microbiol.* 8 1667–1673. 10.1111/j.1462-2920.2006.01052.x 16913926

[B11] Ettensohn C. A. Wray G. Wessel G. M. (2004). *Development of sea urchins, ascidians, and other invertebrate deuterostomes: Experimental approaches*, Vol. 74. Houston, TX: Gulf Professional Publishing.

[B12] Fan C. Liu S. Dai W. He L. Xu H. Zhang H. (2023). Characterization of Vibrio mediterranei isolates as causative agents of vibriosis in marine bivalves. *Microbiol. Spectr.* 11:e04923-22. 10.1128/spectrum.04923-22 36728415 PMC10101119

[B13] Fera M. T. Maugeri T. L. Gugliandolo C. Beninati C. Giannone M. La Camera E. (2004). Detection of *Arcobacter* spp. in the coastal environment of the Mediterranean Sea. *Appl. Environ. Microbiol.* 70 1271–1276. 10.1128/AEM.70.3.1271-1276.2004 15006743 PMC368354

[B14] Huq A. Haley B. J. Taviani E. Chen A. Hasan N. A. Colwell R. R. (2012). Detection, isolation, and identification of *Vibrio cholerae* from the environment. *Curr. Prot. Microbiol.* 26 6A–5A. 10.1002/9780471729259.mc06a05s26 22875567 PMC3461827

[B15] Jurkevitch E. (2006). Isolation and classification of *Bdellovibrio* and like organisms. *Curr. Prot. Microbiol.* 7:7B. 10.1002/9780471729259.mc07b01s00 18770593

[B16] Kongrueng J. Pimonsri Mitraparp-arthorn P. Bangpanwimon K. Robins W. Vuddhakul V. Mekalanos J. (2017). Isolation of *Bdellovibrio* and like organisms and potential to reduce acute hepatopancreatic necrosis disease caused by *Vibrio parahaemolyticus*. *Dis. Aquat. Organ.* 124 223–232. 10.3354/dao03120 28492178

[B17] Laishram M. Rathlavath S. Lekshmi M. Kumar S. Nayak B. B. (2016). Isolation and characterization of *Arcobacter* spp. from fresh seafood and the aquatic environment. *Int. J. Food Microbiol.* 232 87–89. 10.1016/j.ijfoodmicro.2016.05.018 27261768

[B18] Li H. Liu C. Chen L. Zhang X. Cai J. (2011). Biological characterization of two marine *Bdellovibrio*-and-like organisms isolated from Daya Bay of Shenzhen, China and their application in the elimination of *Vibrio parahaemolyticus* in oyster. *Int. J. Food Microbiol.* 151 36–43. 10.1016/j.ijfoodmicro.2011.07.036 21899909

[B19] Liu M. Wei G. Lai Q. Huang Z. Li M. Shao Z. (2023). Genomic and metabolic insights into the first host-associated isolate of *Psychrilyobacter*. *Microbiol. Spectr.* 11:e03990-22. 10.1128/spectrum.03990-22 37754757 PMC10580919

[B20] Lu J. Li X. Qiu Q. Chen J. Xiong J. (2022). Gut interkingdom predator-prey interactions are key determinants of shrimp health. *Aquac.* 546:737304. 10.1016/j.aquaculture.2021.737304

[B21] Magoč T. Salzberg S. L. (2011). FLASH: Fast length adjustment of short reads to improve genome assemblies. *Bioinformatics* 27 2957–2963. 10.1093/bioinformatics/btr507 21903629 PMC3198573

[B22] Martinez-Albores A. Lopez-Santamarina A. Rodriguez J. A. Ibarra I. S. Mondragon A. D. C. Miranda J. M. (2020). Complementary methods to improve the depuration of bivalves: A review. *Foods* 9:129. 10.3390/foods9020129 31991702 PMC7074382

[B23] Mookherjee A. Jurkevitch E. (2022). Interactions between *Bdellovibrio* and like organisms and bacteria in biofilms: Beyond predator–prey dynamics. *Environ. Microbiol.* 24 998–1011. 10.1111/1462-2920.15844 34816563

[B24] Morejón I. F. B. González A. Ferrús M. A. (2017). Detection, identification, and antimicrobial susceptibility of *Arcobacter* spp. isolated from shellfish in Spain. *Foodborne Path. Dis.* 14 238–243. 10.1089/fpd.2016.2202 28121468

[B25] Mottola A. Bonerba E. Figueras M. J. Pérez-Cataluña A. Marchetti P. Serraino A. (2016). Occurrence of potentially pathogenic *arcobacter*s in shellfish. *Food Microbiol.* 57 23–27. 10.1016/j.fm.2015.12.010 27052698

[B26] Musella M. Wathsala R. Tavella T. Rampelli S. Barone M. Palladino G. (2020). Tissue-scale microbiota of the Mediterranean mussel (*Mytilus galloprovincialis*) and its relationship with the environment. *Sci. Total Environ.* 717:137209. 10.1016/j.scitotenv.2020.137209 32084687

[B27] Najnine F. Cao Q. Zhao Y. Cai J. (2020). “Antibacterial activities of *Bdellovibrio* and like organisms in aquaculture,” in *The ecology of predation at the microscale*, eds Jurkevitch E. Mitchell R. J. (Berlin: Springer), 89–96.

[B28] Odeyemi O. A. Burke C. M. Bolch C. C. Stanley R. (2019). Spoilage microbial community profiling by 16S rRNA amplicon sequencing of modified atmosphere packaged live mussels stored at 4°C. *Food Res. Int.* 121 568–576. 10.1016/j.foodres.2018.12.017 31108782

[B29] Ottaviani D. Pieralisi S. Chierichetti S. Rocchegiani E. Hattab J. Mosca F. (2020). *Vibrio parahaemolyticus* control in mussels by a *Halobacteriovorax* isolated from the Adriatic Sea. *Italy. Food Microbiol.* 92:10360. 10.1016/j.fm.2020.103600 32950141

[B30] Parisi A. Addante N. Montagna C. Normanno G. Dambrosio A. Quaglia N. C. (2004). Qualità igienica e presenza di vibrioni in ostriche, vongole e modiole [Hygienic quality and presence of vibrios in oysters, clams, and modiola]. *Ind. Alim.* 43 1–5. Italian Available online at: https://hdl.handle.net/11369/89279

[B31] Pelikan C. Wasmund K. Glombitza C. Hausmann B. Herbold C. W. Flieder M. (2021). Anaerobic bacterial degradation of protein and lipid macromolecules in subarctic marine sediment. *ISME J.* 15 833–847. 10.1038/s41396-020-00817-6 33208892 PMC8027456

[B32] Pérez-Cataluña A. Salas-Massó N. Diéguez A. L. Balboa S. Lema A. Romalde J. L. (2018). Revisiting the taxonomy of the genus *Arcobacter*: Getting order from the chaos. *Front. Microbiol.* 9:364252. 10.3389/fmicb.2018.02077 30233547 PMC6131481

[B33] Rathlavath S. Kumar S. Nayak B. B. (2017). Comparative isolation and genetic diversity of *Arcobacter* sp. from fish and the coastal environment. *Lett. Appl. Microbiol.* 65 42–49. 10.1111/lam.12743 28394467

[B34] Richard G. P. Fay J. P. Dickens K. A. Parent M. A. Soroka D. S. Boyd E. F. (2012). Predatory bacteria as natural modulators of *Vibrio parahaemolyticus* and *Vibrio vulnificus* in seawater and oysters. *Appl. Environ. Microbiol.* 78 7455–7466. 10.1128/AEM.01594-12 22904049 PMC3457099

[B35] Richard M. Rolland J. L. Gueguen Y. de Lorgeril J. Pouzadoux J. Mostajir B. (2021). In situ characterisation of pathogen dynamics during a Pacific oyster mortality syndrome episode. *Mar. Environ. Res.* 165:105251. 10.1016/j.marenvres.2020.105251 33548594

[B36] Romalde J. L. Diéguez A. L. Lasa A. Balboa S. (2014). New *Vibrio* species associated to molluscan microbiota: A review. *Front. Microbiol.* 4:71650. 10.3389/fmicb.2013.00413 24427157 PMC3877837

[B37] Rubiolo J. A. Lozano-Leon A. Rodriguez-Souto R. Fol Rodriguez N. Vieytes M. R. Botana L. M. (2018). The impact of depuration on mussel hepatopancreas bacteriome composition and predicted metagenome. *Antonie van Leeuwenhoek* 111 1117–1129. 10.1007/s10482-018-1015-y 29340947

[B38] Salas-Massó N. Andree K. B. Furones M. D. Figueras M. J. (2016). Enhanced recovery of *Arcobacter* spp. using NaCl in culture media and re-assessment of the traits of *Arcobacter marinus* and *Arcobacter halophilus* isolated from marine water and shellfish. *Sci. Total Environ.* 566 1355–1361. 10.1016/j.scitotenv.2016.05.197 27282494

[B39] Sanger F. Nicklen S. Coulson A. R. (1977). DNA sequencing with chain-terminating inhibitors. *Proc. Natl. Acad. Sci. U S A.* 74 5463–5467. 10.1073/pnas.74.12.5463 271968 PMC431765

[B40] Santibáñez P. Romalde J. Maldonado J. Fuentes D. Figueroa J. (2022). First characterization of the gut microbiome associated with *Mytilus chilensis* collected at a mussel farm and from a natural environment in Chile. *Aquaculture* 548:737644. 10.1016/j.aquaculture.2021.737644

[B41] Santoro M. Viscardi M. Boccia F. Borriello G. Lucibelli M. G. Auriemma C. (2020). Parasite load and STRs genotyping of *Toxoplasma gondii* isolates from Mediterranean mussels (*Mytilus galloprovincialis*) in southern Italy. *Front. Microbiol.* 11:355. 10.3389/fmicb.2020.00355 32210944 PMC7066981

[B42] Schoinas K. Konstantou V. Bompou E. Floros G. Chatziplis D. Imsiridou A. (2023). Microbiome profile of the Mediterranean mussel (*Mytilus galloprovincialis*) from Northern Aegean Sea (Greece) culture areas, based on a 16S rRNA next generation sequencing approach. *Diversity* 15:463. 10.3390/d15030463

[B43] Serracca L. Gallo F. Magone L. Preparo M. Ercolini C. Orlandi M. (2007). Caratterizzazione biochimica e tossicologica di Vibrio patogeni in prodotti ittici [Biochemical and toxicological characterization of pathogenic Vibrio in seafood]. *Ind. Alim.* 46 881–886. Italian

[B44] Serratore P. Ciulli S. Piano A. Cariani A. (2014). “Criticism of the purification process of bivalve shellfish. Literature review and our industrial research experiences,” in *Shellfish, human consumption health implication and conservation concerns*, ed. Hay R. M. (Hauppauge, NY: Nova Science Publishers Inc), 1–50. Available online at: http://www.scopus.com/inward/record.url?eid=2-s2.0-35448995240&partnerID=MN8TOARS

[B45] Sferlazzo G. Meloni D. Lamon S. Marceddu M. Mureddu A. Consolati S. G. (2018). Evaluation of short purification cycles in naturally contaminated Mediterranean mussels (*Mytilus galloprovincialis*) harvested in Sardinia (Italy). *Food Microbiol.* 74 86–91. 10.1016/j.fm.2018.03.007 29706341

[B46] Sharp J. H. Clements K. Diggens M. McDonald J. E. Malham S. K. Jones D. L. (2021). *E. coli* is a poor end-product criterion for assessing the general microbial risk posed from consuming norovirus contaminated shellfish. *Front. Microbiol.* 12:608888. 10.3389/fmicb.2021.608888 33679634 PMC7933002

[B47] Suffredini E. Mioni R. Mazzette R. Bordin P. Serratore P. Fois F. (2014). Detection and quantification of *Vibrio parahaemolyticus* in shellfish from Italian production areas. *Int. J. Food Microbiol.* 184 14–20. 10.1016/j.ijfoodmicro.2014.04.016 24810197

[B48] Ter Ü Gürkan S. E. Gürkan M. Kunili I. E. Aksoy E. (2024). Pathological and oxidative stress responses of *Mytilus galloprovincialis* to *Vibrio mediterranei* infection: An in vivo challenge. *Fish Shellfish Immunol.* 154:109889. 10.1016/j.fsi.2024.109889 39250984

[B49] Vezzulli L. Stagnaro L. Grande C. Tassistro G. Canesi L. Pruzzo C. (2018). Comparative 16SrDNA gene-based microbiota profiles of the Pacific oyster (*Crassostrea gigas*) and the Mediterranean mussel (*Mytilus galloprovincialis*) from a shellfish farm (Ligurian Sea. *Italy). Microb. Ecol.* 75 495–504. 10.1007/s00248-017-1051-6 28803409

[B50] Wang H. Qi M. Cutler A. J. (1993). A simple method of preparing plant samples for PCR. *Nucl Acids Res.* 21 4153–4154. 10.1093/nar/21.17.4153 8371994 PMC310032

[B51] Wang J.-S. Peng L.-H. Guo X.-P. Yoshida A. Osatomi K. Li Y.-F. (2019). Complete genome of *Pseudoalteromonas atlantica* ECSMB14104, a Gammaproteobacterium inducing mussel settlement. *Mar. Genom.* 46 54–57. 10.1016/j.margen.2018.11.005

[B52] Weisburg W. G. Barns S. M. Pelletier D. A. Lane D. J. (1991). 16S ribosomal DNA amplification for phylogenetic study. *J. Bacteriol.* 173 697–703. 10.1128/jb.173.2.697-703.1991 1987160 PMC207061

[B53] Williams H. Lymperopoulou D. Athar R. (2016). *Halobacteriovorax*, an underestimated predator on bacteria: Potential impact relative to viruses on bacterial mortality. *ISME J.* 10 491–499. 10.1038/ismej.2015.129 26251870 PMC4737939

[B54] Zhang H. Zou X. Hu J. Liu S. Fan C. Dai W. (2025). Genomic insights into mechanism underlying virulence variations between *Vibrio mediterranei* strains different in pathogenicity toward bivalves. *Aquaculture* 605:742524. 10.1016/j.aquaculture.2025.742524

[B55] Zhang L. Guo L. Cui Z. Ju F. (2024). Exploiting predatory bacteria as biocontrol agents across ecosystems. *Trends Microbiol.* 32 398–409. 10.1016/j.tim.2023.10.005 37951768

[B56] Zhang X. Alter T. Gölz G. (2019). Characterization of *Arcobacter* spp. isolated from retail seafood in Germany. *Food Microbiol.* 82 254–258. 31027781 10.1016/j.fm.2019.02.010

