# Supplementary material for: Impact of predation on the bacterial community structure of Mediterranean mussels during depuration
Source: Front Microbiol. 2025 Nov 6;16:1647926. doi: 10.3389/fmicb.2025.1647926 (PMC12631426; doi:10.3389/fmicb.2025.1647926)
Supplement: Supplementary file 1 [file Supplementary_file_1.docx]

Supplementary Material


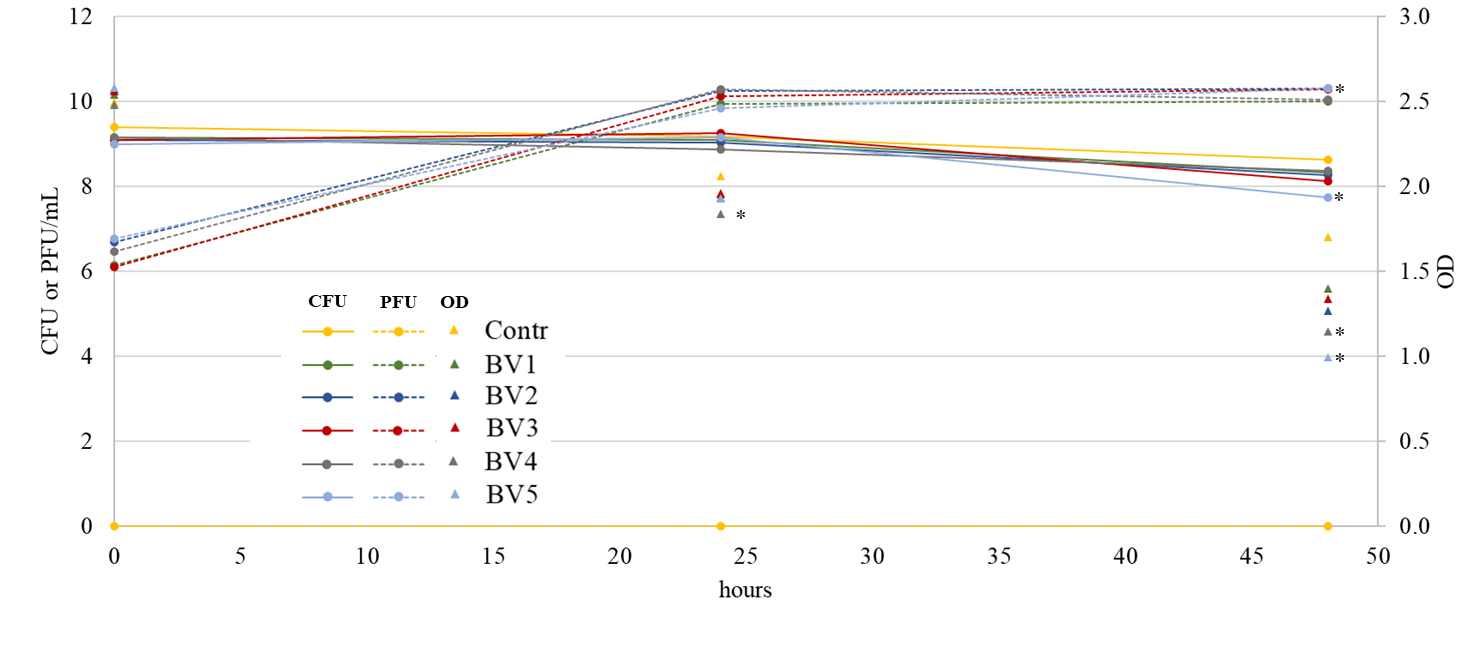


**Supplementary Figure 1.** Lytic activity of 5 predatory strains (BV1, BV2, BV3, BV4, and BV5) against *V. mediterranei* VM6 monitored by OD600 reduction (triangles), CFU/mL of the prey (continuous lines), and PFU/mL of the predators (dashed lines) at time 0 and after 24 and 48 h of incubation.

**
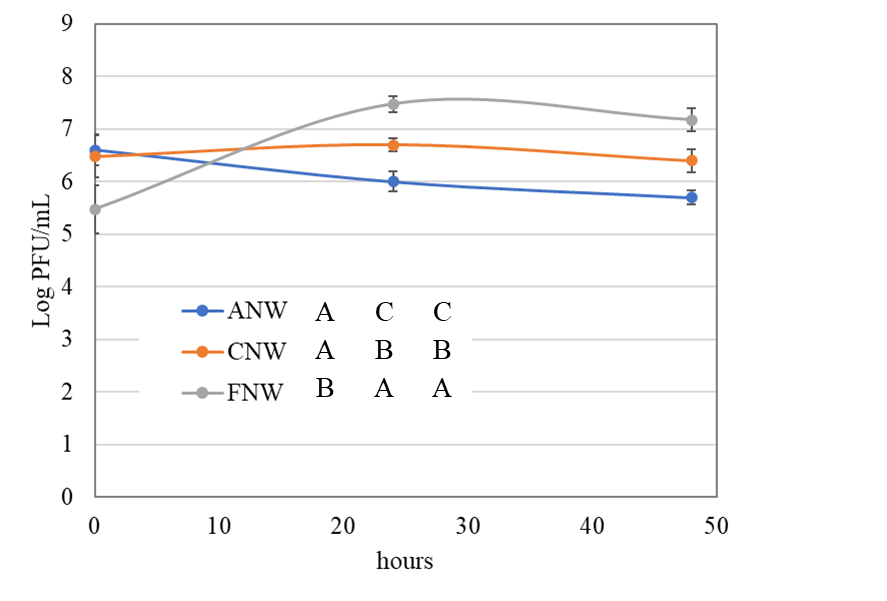
**

**Supplementary Figure 2.** BALOs (PFU/mL) at time 0 and after 24 and 48 hours in ASW (NW) from the first depuration experiment carried out with non-depurated mussels. Trial A: prey and predator; Trial C: only predator; Trial F: prey and predator in ASW without mussels. For data with the same letter, differences between trials are not statistically significant (*p* < 0.05).


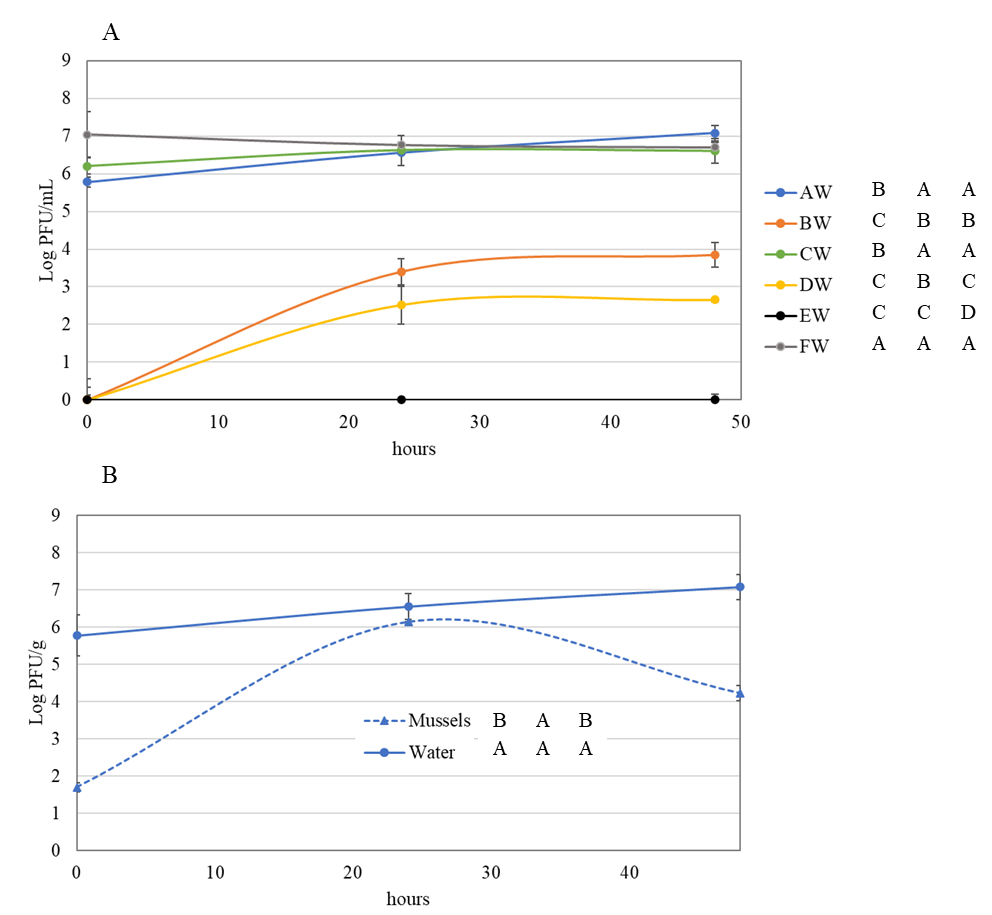


**Supplementary Figure 3. Panel (A):** BALOs (PFU/mL) at time 0 and after 24 and 48 hours in ASW (W) from the second depuration experiment carried out with depurated mussels. Trial A: prey and predator; Trial B: only prey; Trial C: only predator; Trial E and F: prey and prey plus predator in ASW without mussels. **Panel (B):** BALOs (PFU/mL) at 0 and after 24 and 48 hours in mussels compared with predator PFU counts in water (AW).

For data with the same letter, differences between trials are not statistically significant (*p* < 0.05).


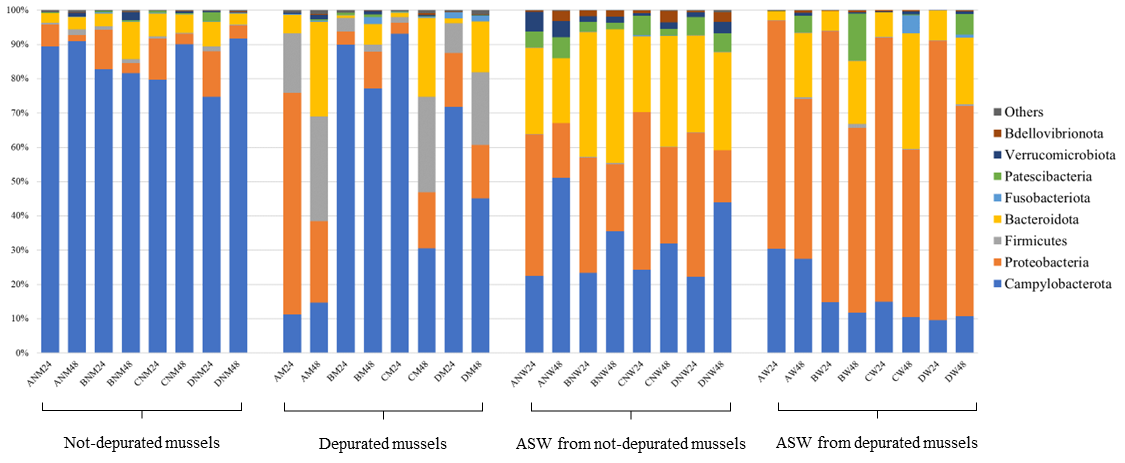


**Supplementary Figure 4.** Barplots showing the mean relative abundance of bacterial *phyla* in not-depurated (NM) and depurated (M) mussels and in the depuration waters (NW and W) after 24 and 48 hours of depuration. Only taxa with a mean relative abundance > 1% are plotted.

**Supplementary Table 1.** Heterotrophic microflora counts on WPCA in non-depurated mussels (NM) and water (NW) in the four trials A, B, C, and D. Data are reported as Log CFU/mL or g (mean±sd)

| Trial | Time (hours) | | | |
| --- | --- | --- | --- | --- |
|  | 0 | 4 | 24 | 48 |
| NMA | 3.43±0.60 | 3.92±0.19 | 4.03±0.24 | 3.82±0.14 |
| NMB | 3.21±0.01 | 4.07±0.44 | 4.12±0.54 | 3.70±0.02 |
| NMC | 3.51±0.40 | 3.96±0.34 | 4.96±0.15 | 3.92±0.11 |
| NMD | 3.63±0.60 | 3.96±0.01 | 4.18±0.02 | 3.77±0.23 |
| NWA | 3.21±0.23 | 3.34±0.17 | 3.92±0.44 | 2.77±0.34 |
| NWB | 3.28±0.14 | 4.00±0.34 | 3.92±0.19 | 3.15±0.01 |
| NWC | 3.18±0.14 | 4.03±0.25 | 3.62±0.35 | 2.77±0.54 |
| NWD | 3.15±0.02 | 4.03±0.40 | 3.52±0.21 | 2.77±0.15 |

**Supplementary Table 2.** Heterotrophic microflora (WPCA), *Enterobacteriaceae* (VRBGA), *Coli-aerogenes* group (VRBLA), *E. coli* (TBX), and enterococci (Slanetz & Barley) counts in depurated mussels (M) and water (W) in the four trials A, B, C, and D (Log CFU/mL or g ± sd).

| Media | Time (hours) | | | | |
| --- | --- | --- | --- | --- | --- |
|  |  | 0 | 4 | 24 | 48 |
| PCA | AM | 5.14±0.02 | 4.66±0.88 | 4.70±0.15 | 4.77±0.60 |
|  | BM | 5.24±0.14 | 4.66±0.33 | 4.49±0.49 | 5.03±0.41 |
|  | CM | 5.00±0.23 | 4.73±0.18 | 4.52±0.31 | 5.32±0.21 |
|  | DM | 4.66±0.42 | 4.65±0.39 | 4.75±0.48 | 5.40±0.32 |
|  | AW | 2.02±0.11 | 3.64±0.09 | 3.92±0.25 | 4.28±0.09 |
|  | BW | 1.67±0.09 | 3.56±0.01 | 4.03±0.11 | 5.20±0.11 |
|  | CW | 2.00±0.00 | 3.67±0.07 | 4.49±0.07 | 4.92±0.15 |
|  | DW | 1.86±0.31 | 3.46±0.17 | 4.52±0.29 | 5.32±0.01 |
|  |  |  |  |  |  |
| VRBGA | AM | 1.08±0.40 | 0.30±0.48 | 1.20±0.13 | 1.85±0.51 |
|  | BM | 0.94±0.09 | 1.13±0.03 | 1.53±0.09 | 1.08±0.50 |
|  | CM | 1.11±0.53 | 0.00±0.26 | 2.23±0.44 | 1.96±0.30 |
|  | DM | 1.09±0.07 | 1.00±0.18 | 2.28±0.62 | 2.27±0.24 |
|  | AW | - | 0.30±0.03 | 0.78±0.05 | 0.48±0.04 |
|  | BW | - | 0.70±0.00 | 0.70±0.04 | 0.70±0.18 |
|  | CW | - | 0.78±0.19 | 0.60±0.16 | 1.93±0.11 |
|  | DW | - | 1.20±0.20 | 1.04±0.01 | 2.25±0.09 |
|  |  |  |  |  |  |
| VRBLA | AM | 1.70±0.35 | 1.72±0.07 | 2.11±0.44 | 1.74±0.44 |
|  | BM | 1.58±0.33 | 1.83±0.64 | 2.25±0.26 | 1.18±0.09 |
|  | CM | 1.62±0.13 | 1.04±0.42 | 2.38±0.17 | 2.18±0.16 |
|  | DM | 1.08±0.08 | 1.00±0.03 | 2.60±0.11 | 2.41±0.11 |
|  | AW | - | 1.20±0.09 | 2.30±0.04 | 1.28±0.08 |
|  | BW | - | 1.41±0.00 | 1.26±0.31 | 1.36±0.00 |
|  | CW | - | 1.61±0.12 | 1.04±0.16 | 2.18±0.32 |
|  | DW | - | 1.70±0.25 | 1.61±0.03 | 2.47±0.17 |
|  |  |  |  |  |  |
| TBX | AM | Nd | Nd | Nd | Nd |
|  | BM | Nd | 0.30±0.00 | Nd | Nd |
|  | CM | Nd | Nd | Nd | Nd |
|  | DM | Nd | Nd | 0.30±0.01 | Nd |
|  | AW | - | Nd | Nd | Nd |
|  | BW | - | Nd | Nd | Nd |
|  | CW | - | Nd | Nd | Nd |
|  | DW | - | Nd | 0.30±0.00 | 0.30±0.01 |
|  |  |  |  |  |  |
| SB | AM | Nd | Nd | Nd | Nd |
|  | BM | Nd | 0.30±0.21 | Nd | 2.22±0.13 |
|  | CM | Nd | Nd | Nd | Nd |
|  | DM | Nd | 0.30±0.15 | Nd | Nd |
|  | AW | - | Nd | Nd | Nd |
|  | BW | - | Nd | Nd | Nd |
|  | CW | - | Nd | Nd | 0.30±0.00 |
|  | DW | - | Nd | Nd | Nd |
